# Unexpected Presentations of Bradycardia, Renal Failure, Atrioventricular Nodal Blockade, Shock, and Hyperkalemia (BRASH) Syndrome: A Report of Two Cases

**DOI:** 10.7759/cureus.58900

**Published:** 2024-04-24

**Authors:** Humail Patel, Justin Lin, Lilly Hou, Lawrence Belletti

**Affiliations:** 1 Internal Medicine, Northwell Health, New Hyde Park, USA

**Keywords:** cardiogenic shock, renal failure, hyperkalemia, bradycardia, brash syndrome

## Abstract

Bradycardia, renal failure, atrioventricular nodal blockade, shock, and hyperkalemia syndrome is an underrecognized phenomenon in which renal injury leads to hyperkalemia and inadequate clearance of atrioventricular nodal-blocking agents. The compounding effect of both insults can lead to a bradyarrhythmia that, in severe cases, can rapidly progress to cardiogenic shock. The degree of resulting pathology is usually out of proportion to either insulting agent given that there is a synergistic effect. Treatment strategies for this condition are not entirely clear, but it appears as if these patients often do not warrant aggressive interventions and can be managed medically. We report two cases with early recognition and simple medical management with resulting favorable outcomes.

## Introduction

Bradycardia, renal failure, atrioventricular nodal blockade, shock, and hyperkalemia (BRASH) syndrome is a constellation of symptoms that characterize cardiogenic shock in the context of its precipitating and conjunctive factors. In BRASH syndrome, it is hypothesized that an acute renal injury can result in reduced clearance of atrioventricular (AV) nodal-blocking agents and potassium. This synergistic, yet detrimental effect of concomitant cardiac dysconduction can result in catastrophic sequelae disproportionate to either level of the expected insulting agent alone. Patients typically present with bradycardia, often with a junctional rhythm and sinus arrest identified on an electrocardiogram (ECG) [1]. The resulting clinical manifestations can vary from asymptomatic bradycardia to severe cardiogenic shock. Fortunately, given the treatable precipitating factors, these patients seldom require pacing or aggressive interventions and their bradyarrhythmia often resolves with intracellular shifting of potassium [2].

BRASH syndrome continues to be poorly understood primarily due to underreporting and a lack of awareness. In clinical settings, the lack of recognition of this syndrome may account for poor outcomes and the high in-hospital mortality associated with it [3]. The management strategy, given the pathophysiology outlined above, may differ from that of hyperkalemia or that of AV nodal blocker toxicity independently given the concomitant effect [2]. Cases of BRASH syndrome are often diagnosed despite normal and even low doses of AV nodal-blocking agents [1]. As such, a high index of suspicion and early identification of this condition is critical in guiding management. Herein, we present two cases of BRASH syndrome precipitated by very common and seemingly benign-appearing etiologies of acute kidney injury with fortunately favorable outcomes.

## Case presentation

Case 1: BRASH syndrome precipitated by postoperative urinary retention

**Figure 1:**
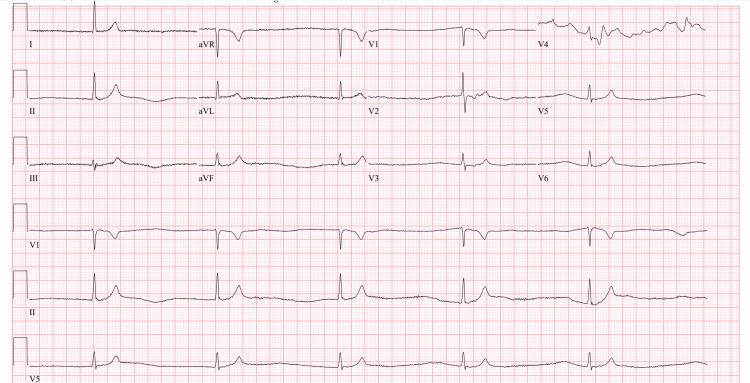
Initial electrocardiogram revealing junctional bradycardia at a rate of 33 beats per minute.

**Figure 2:**
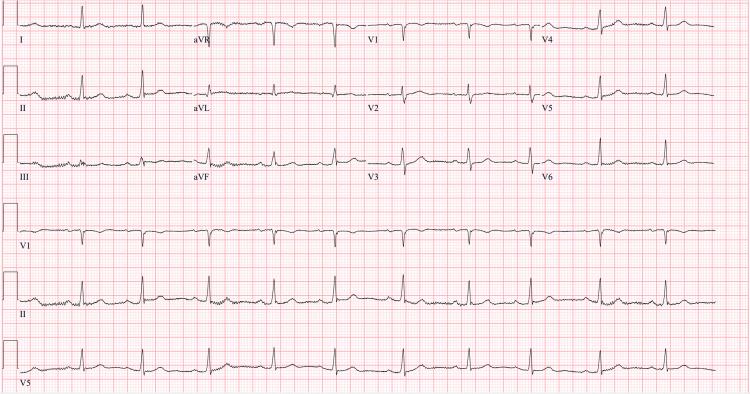
Repeat electrocardiogram several hours after shifting of hyperkalemia, revealing normal sinus rhythm at a rate of 64 beats per minute.

An 81-year-old female with heart failure with preserved ejection fraction, chronic obstructive pulmonary disease, hypertension, hyperlipidemia, stage three chronic kidney disease, and a recent open reduction and internal fixation for an ankle fracture two weeks prior presented from her rehabilitation facility for fatigue and lightheadedness for several hours. Upon assessment at the facility, the patient was found to be bradycardic with a reported heart rate between 30 and 40 beats per minute and significant hyperkalemia. The patient was given an unknown amount of sodium polystyrene sulfonate (Kayexalate) and transferred to the emergency department (ED). In the ED, her bradycardia persisted with her heart rate dropping to as low as 32 beats per minute while remaining normotensive. She appeared fatigued and lethargic on the examination but remained awake and alert. Initial laboratory workup was remarkable for hyponatremia, hyperkalemia, metabolic acidosis, acute kidney injury, and transaminitis (Table 1). ECG was notable for junctional bradycardia with a rate of 33 beats per minute with sinus node arrest (Figure 1). The patient was treated with 2 g of intravenous calcium gluconate, 5 units of regular insulin, and a dose of albuterol via a nebulizer. She did not undergo any transcutaneous pacing and was admitted to the cardiac intensive care unit for further monitoring. A review of the patient’s cardiac medications revealed that she was taking carvedilol and spironolactone. Shortly after intracellular shifting of her potassium, the patient returned to a normal sinus rhythm at about 60 beats per minute (Figure 2). The patient was subsequently transferred to the general medical floor where her course was complicated by acute cystitis with persistently elevated serum creatinine. Further investigation revealed bilateral hydronephrosis and elevated post-void residual bladder volumes. The patient underwent placement of a Foley catheter and was scheduled for outpatient urology follow-up for presumed postoperative urinary retention. The patient’s carvedilol and spironolactone were discontinued upon discharge.

**Table 1:** Lab values at presentation.

	Value at presentation	Reference range
Sodium	129 mmol/L	135–145 mmol/L
Potassium	6.6 mmol/L	3.5–5.3 mmol/L
Creatinine	2.49 mg/dL (baseline 1.1 mg/dL)	0.50–1.30 mg/dL
HCO_3_	19 mmol/L	22–31 mmol/L
Aspartate aminotransferase	381 U/L	10–40 U/L
Alanine aminotransferase	353 U/L	10–45 U/L
Lactate	1.8 mmol/L	0.5–2.0 mmol/L

Case 2: BRASH syndrome precipitated by COVID-19 infection

**Figure 3:**
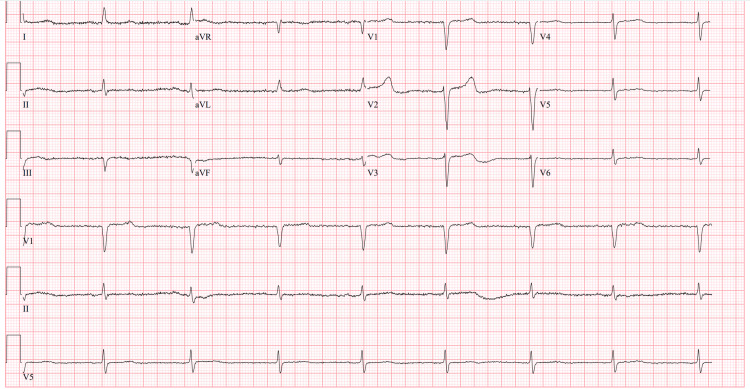
Initial electrocardiogram revealing junctional bradycardia at a rate of 49 beats per minute.

A 99-year-old female with hypertension, gout, and chronic anemia presented to the ED after testing positive for COVID-19 on a home test. She also reported frequent mechanical falls over the past few days, along with generalized weakness. Upon arrival at the ED, she was found to be hypothermic to 32.8°C, hypoxic with an oxygen saturation of 88% on room air, and bradycardic with a heart rate of 40-45 beats per minute. Her physical examination revealed a grade III/VI systolic ejection murmur and bilateral lower extremity pitting edema. Initial laboratory workup was remarkable for hyponatremia, hyperkalemia, elevated blood urea nitrogen, and acute kidney injury; serum lactate was within normal limits (Table 2). Her chest radiograph did not show any evidence of pneumonia or other lung pathology. Her ECG revealed junctional bradycardia at a rate of 49 beats per minute (Figure 3). The patient received 2 g of intravenous calcium gluconate and 5 units of regular insulin. The patient did not undergo any transcutaneous pacing and was admitted to the medical floor for further management. Upon review of her home medications, the patient was found to be prescribed losartan and carvedilol, both of which were discontinued. Shortly after intracellular shifting of her potassium, the patient was noted to spontaneously return to a normal sinus rhythm at a rate of 70-80 beats per minute, as noted on telemetry monitoring. For further workup of her hypothermia, the patient underwent testing of an AM cortisol as well as a thyroid panel, both of which were within normal limits. The patient’s acute kidney injury improved with gentle intravenous fluid resuscitation and encouragement of increased oral intake, suggesting a prerenal etiology. The patient was eventually discharged home with instructions to discontinue her beta-blocker.

**Table 2:** Lab values at presentation.

	Value at presentation	Reference range
Sodium	130 mmol/L	135–145 mmol/L
Potassium	6.6 mmol/L	3.5–5.3 mmol/L
Blood urea nitrogen	57 mg/dL	7–23 mg/dL
Creatinine	1.61 mg/dL (baseline 1.2 mg/dL)	0.50–1.30 mg/dL
Lactate	1.2 mmol/L	0.5–2.0 mmol/L

## Discussion

The pathophysiology behind BRASH syndrome appears to be driven by several concomitant factors. It involves patients who are already on existing AV nodal-blocking agents, most commonly beta-blockers, at baseline. Acute renal injury, from any etiology, can lead to inadequate clearance of AV nodal-blocking agents and potassium. The synergistic effect of hyperkalemia and increased levels of AV nodal-blocking agents in the serum can result in significant bradycardia. BRASH syndrome is also often seen in patients on angiotensin-converting enzyme inhibitors or angiotensin receptor blockers (ARBs) as these medications may exacerbate both their renal injury and hyperkalemia [3]. This bradycardia leads to decreased forward perfusion of the kidneys, thereby worsening renal failure. This creates a vicious cycle that can rapidly progress to multiorgan failure, with a reported in-hospital mortality of nearly 6% [2].

Interestingly, given that this pathologic effect is a combination of both hyperkalemia and AV nodal blockade, severe bradycardia can be seen at lower doses of both offending agents than typically expected. The majority of these patients present with non-severe hyperkalemia (<6.5 mmol/L) but experience bradycardia-induced shock [2]. As such, the typical ECG findings associated with hyperkalemia, including peaked T waves, lengthening of the PR interval, shortened QT interval, and widening of the QRS complex, are often not appreciated [4]. Prior studies have reported that the most common ECG findings are junctional bradycardia, sinus bradycardia, or complete heart block [5]. Despite the potential severity of this bradyarrhythmia, most patients do not require pacing as the correction of the underlying hyperkalemia and management of their acute renal injury can lead to rapid improvement in their symptoms. All insulting agents, whether calcium channel blockers, beta-blockers, or alternative AV nodal blocking agents, should be held both during the index hospitalization and definitively thereafter to prevent recurrence.

As demonstrated in the cases above, acute kidney injury is often thought to be the preceding factor. In many elderly patients, the degree of insult needed to precipitate acute kidney injury may not be substantial. However, once the kidney injury and resulting hyperkalemia develop, the concomitant bradycardia leads to decreased perfusion of the kidneys, rapidly worsening the injury, likely via acute tubular necrosis [6]. This, in turn, leads to further worsening of the hyperkalemia and decreased clearance of the AV nodal-blocking agent, creating a detrimental cycle of events [7]. In our first case, despite rehabilitating appropriately from her orthopedic surgery, the patient had mild urinary retention, a very common postoperative complication in the elderly, resulting in hydronephrosis and kidney injury. In the setting of existing AV nodal blockade (carvedilol in this case), this triggered the severe cycle of BRASH syndrome. Fortunately, her bradyarrhythmia resolved shortly after intracellular shifting of potassium with insulin and albuterol. In the second case, an elderly female presented with fatigue and lethargy presumably secondary to COVID-19. As such, her decreased oral intake and functional status precipitated a mild acute kidney injury. This, in conjunction with her existing AV nodal blockade and ARB, likely led to her presentation of BRASH syndrome. The bradyarrhythmia also possibly contributed to her reported unexplained frequent falls at home. Prompt treatment of the hyperkalemia led to a quick resolution of her bradyarrhythmia. In both cases, AV nodal blockade was discontinued indefinitely. Neither patient required pacing or emergent management of the bradycardia itself with pacing or vasopressors given their relative hemodynamic stability.

## Conclusions

BRASH syndrome continues to be an underreported and underdiagnosed entity despite its significant mortality. It often presents as symptomatic bradycardia in the setting of an acute renal injury that precipitates decreased clearance of AV nodal-blocking agents and hyperkalemia. Our cases highlight the importance of considering this diagnosis when bradycardia and hyperkalemia are seen in the setting of preexisting AV nodal blockade. Fortunately, as demonstrated in our cases, many patients often respond to simple medical management and intracellular shifting of their hyperkalemia. Prompt recognition of this condition is essential not only to guide management but also to avoid unnecessary invasive and aggressive interventions such as pacemaker implantation or the initiation of vasoactive or inotropic medications.
